# Analysis of the HPLC Fingerprint and QAMS for Sanhuang Gypsum Soup

**DOI:** 10.1155/2018/5890973

**Published:** 2018-07-02

**Authors:** Yi Peng, Minghui Dong, Jing Zou, Zhihui Liu

**Affiliations:** Department of Pharmacy, Nanjing University of Traditional Chinese Medicine Affiliated Hospital, Nanjing 210029, China

## Abstract

A valid and encyclopaedic evaluation method for assessing the quality of Sanhuang Gypsum Soup (SGS) has been set up based on analysis of high-performance liquid chromatography (HPLC) fingerprint combined with the quantitative analysis of multicomponents by single marker (QAMS) method, hierarchical cluster analysis (HCA), and similarity analysis. 20 peaks of the common model were obtained and used for the similarity analysis and HCA analysis. Berberine was selected as an internal reference, and the relative correction factors of mangiferin, geniposide, liquiritin, epiberberine, coptisine, baicalin, palmatine, harpagosid, wogonoside, cinnamic acid, cinnamic aldehyde, baicalein, glycyrrhizic acid, and wogonin were established. The accuracy of quantitative analysis of multicomponents by the single-marker method was verified by comparing the contents of the fourteen components calculated by the external standard method with those of the quantitative analysis of multicomponents by the single-marker method. No significant difference was found in the quantitative results of the established quantitative analysis of multicomponents by a single-marker method and an external standard method. In summary, these methods were applied to evaluate the quality of SGS successfully. As a result, these evaluation methods have great potential to be widely used in the quality control of traditional Chinese medicines (TCM).

## 1. Introduction

Perspiration is a considerable physiological phenomenon to maintain and control body temperature. Excessive sweat secretion can cause armpit moisture, resulting in unpleasant body odour, embarrassment, and inconvenience [1, 2]. Hyperhidrosis is an excessive sweating disease which can bring severe psychological burden and affect the quality of life of patients negatively. There are a variety of medical treatments and surgery for treating primary hyperhidrosis. However, the side effects of these drugs include thirst, dry eyes, dizziness, drowsiness, constipation, and urinary retention, which limit the scope of their use. Surgical treatment is mainly applied to patients who are not suitable for the abovementioned methods. Surgery is more traumatized and risky than other treatments. Therefore, surgical operation should be as a second or third option [3]. There is still a paucity of effective nonsurgical therapies. With the development of modern society, the elimination of body odour is given more and more people's attention; this article is about introducing Sanhuang gypsum soup (SGS) which is a significant antiperspirant. Through the use of classical Chinese medicine SGS to regulate the internal environment of the human body, it achieves a good antiperspirant effect with small side and remarkable effects, which make up for deficiency of some medicine and surgery. Every year, more than ten thousands of patients have benefited from SGS by reducing excessive perspiration symptoms. SGS is a hospital preparation of Jiangsu Provincial Hospital of Traditional Chinese Medicine, which consists of coptidis rhizoma, phellodendri chinensis cortex, scutellariae radix, scrophulariae radix, anemarrhenae rhizome, gardeniae fructus, cinnamomi cortex, glycyrrhizae radix et rhizoma preparata cum melle, and gypsum fibrosum. These herbs can be used for treating and could exhibit action on excessive perspiration through anti-inflammatory and antipyretic properties. Studies have shown that SGS has many chemical constituents such as mangiferin, geniposide, coptisine, wogonin, wogonoside, baicalein, baicalinin, and cinnamic aldehyde [4–13] in accordance with herbs and preparations known to be beneficial for the treatment of excessive perspiration through anti-inflammatory properties and so on.

Although the SGS is prepared as a prescription with the combination of these herbs in well-defined formulae, no standard quality control method for this product has been reported up to now. Since the effect of SGS might result from the synergy of multiple components, a reliable, sensitive, and uncomplicated quantitative method based on the diverse constituents is need to be developed.

Our findings have established an HPLC method to evaluate the quality of SGS comprehensively. Due to the variety of components of traditional Chinese medicine preparations, any one of the active ingredients cannot reflect the overall curative effect of traditional Chinese medicine. Therefore, a comprehensive macroscopic analysis will become an inevitable trend. Chromatographic fingerprint analysis with integrated, macroscopic, and “fuzzy” nonlinear characteristics is more adapted to the traditional Chinese medicine theory needs. Under the premise of efficacy, toxicology, and clinical trials which have confirmed safety and efficacy of preparation, we can not only verify the authenticity of the preparation but also determine whether the stability of the quality exists or not along with a practical fingerprint. Unlike the content determination, the fingerprint can provide more informative and useful message than the determination of any single component. The US Food and Drug Administration (FDA) allows applicants to provide product chromatographic fingerprinting information in the phytomedical guidance (Draft for Comment). British Herbal Codex, Ayurvedic Codex, the Canadian Society of Medicinal and Aromatic Plants [14], and the German Society of Medicinal Plants [15] also accept chromatographic fingerprint. One of the first measures that China's State Drug Administration has taken to strengthen the supervision of traditional Chinese medicine injections requires the research on the fingerprint of injections, which has taken into account its necessity and feasibility. It is accepted that preparation of acceptable quality can be exerted on its drug efficacy, what really matters is establishing an accurate and easy method.

Previously, our laboratory has researched on the fingerprints of the existing preparations which have been applied in the control of preparation quality. Also, to make up for the limitations of fingerprint that cannot be quantified accurately, a QAMS method using berberine as the standard was developed and validated for the simultaneous quantitative of 14 components [16]. This strategy can not only reduce the cost of the experiment and time of detection but also be independent of the availability of all the target ingredients [15]. To our knowledge, quality control of herb extracts and botanical ingredient by QAMS have been included both in USP 33-NF and in Ch.P.2010 edition (volume I). Our results showed that no significant difference was found in the results between our established QAMS method and the external standard method. No one has yet studied the fingerprints of SGS; this article first established the SGS fingerprinting method and also used the QAMS method to measure the preparation of 14 kinds of pharmacodynamics components. This method could potentially be applied for the identification of qualitative and quantitative quality of SGS.

This HPLC fingerprint method, therefore, provides a comprehensive platform for quality evaluation of SGS with more chemical information. The combination of HCA and similarity analysis presents the differences and similarities of the HPLC fingerprints. In the meantime, QAMS method was adopted to quantify the main active components by comparing with the external standard method (ESM) in all the SGS samples. Our findings offer a new routine for assessing the quality of TCM.

## 2. Materials and Methods

### 2.1. Chemicals and Reagents

Analysis was applied on three different HPLC systems, including (a) Agilent 1100 series with vacuum degasser (G1322A), quaternary pump (G1311A), autosampler (G1316A), and a ChemStation Workstation with VWD detector; (b) Agilent 1260 series with DAD detector and Agilent ChemStation Workstation; and (c) Waters 2695-2996 series with 2998PDA detector and empower workstation. The chromatographic separation was performed on an AmethyC_18_ (4.6 mm × 250 mm, 5 *μ*m) column, Agilent C_18_ (4.6 mm × 250 mm, 5 *μ*m) column, and HedraC_18_ (4.6 mm × 250 mm, 5 *μ*m) column.

The SPSS software (Edition 2.0) was used for conducting cluster analysis.

BP-211D electronic analytical balance (Germany Sartorius Company) was used to weigh the drugs. Sonicator (SK6200H, Shanghai Branch guided ultrasound instrument Co., Ltd.) was used to help dissolve the sample.

### 2.2. Materials

The batch numbers and origins of eight qualified Chinese herbal pieces of decoction are shown in Table 1. All pieces were purchased from Anhui Concord Pharmaceutical Pieces Co., Ltd. and identified by Professor Zhihui Liu of Nanjing University of Traditional Chinese Medicine.

Fifteen batches of Sanhuang Gypsum Soup were provided by the Department of Pharmacy of Jiangsu Provincial Hospital. Their batch numbers were S1 (1707010), S2 (1704006), S3 (1712019), S4 (1711016), S5 (1704005), S6 (1703004), S7 (1711013), S8 (1711017), S9 (1705007), S10 (1704003), S11 (1704002), S12 (1704001), and S13 (1702015). Each single piece preparations and its negative preparations are made by our laboratory as per the preparation standard process.

### 2.3. Chemical Reagents and Standards

Mangiferin, geniposide, liquiritin, epiberberine, coptisine, baicalin, palmatine, berberine, harpagosid, wogonoside, cinnamic acid, cinnamic aldehyde, baicalein, glycyrrhizic acid, and wogonin were all supplied by Chengdu Mansi Biotechnology Co., Ltd. The purity of each ingredient was greater than 98% as determined by HPLC. Acetonitrile of HPLC grade and formic acid of analytical grade were purchased from Merck (Darmstadt, Germany) and Roe Scientific Inc. (USA). A Milli-Q water (Millipore, Inc., USA) purification system was applied to purify water for the HPLC analysis.

### 2.4. Preparation of the Sample Solution

The sample solution of SGS was precisely absorbed (5 ml) and immersed in 25 mL volumetric flask with methanol. Additional methanol was added to compensate the weight loss after ultrasonic extraction for 30 min. All solutions were filtered through 0.45 *μ*m filter membranes before being injected into the HPLC system precisely.

### 2.5. Reference Solution Preparation

A mixed stock solution containing reference standards was prepared by dissolving weighed samples of each compound in methanol accurately. Then, the stock solutions were diluted to establish the calibration curves based on six appropriate concentrations with the ranges of 2.80–88.70 *μ*g·ml^−1^ for mangiferin, 14.80–472.90 *μ*g·ml^−1^ for geniposide, 3.20–101.00 *μ*g·ml^−1^ for liquiritin, 1.40–44.30 *μ*g·ml^−1^ for epiberberine, 6.40–204.10 *μ*g·ml^−1^ for coptisine, 19.80–632.50 *μ*g·ml^−1^ for baicalin, 1.80–56.80 *μ*g·ml^−1^ for palmatine, 15.30–488.40 *μ*g·ml^−1^ for berberine, 2.60–82.60 *μ*g·ml^−1^ for harpagosid, 4.60–145.82 *μ*g·ml^−1^ for wogonoside, 0.30–9.51 *μ*g·ml^−1^ for cinnamic acid, 0.20–6.34 *μ*g·ml^−1^ for cinnamic aldehyde, 0.50–15.85 *μ*g·ml^−1^ for baicalein, 1.10–34.00 *μ*g·ml^−1^ for glycyrrhizic acid, and 0.30–9.51 *μ*g·ml^−1^ for wogonin.

### 2.6. Chromatographic Procedures

Analytes were separated on a reverse phase C_18_ column (Amethyl-ODS-2 C_18_ column, 250 mm ∗ 4.6 mm ∗ 5 *μ*m).

Mobile phase consists of 0.1% phosphoric acid (A)-acetonitrile (B), gradient elution program was as follows: 0∼2 min, 12% B; 2∼7 min, 12%∼20% B; 7∼17 min, 20%∼25% B; 17∼25 min, 25%∼32% B; 25∼32 min, 32%∼35% B; 32∼45 min, 35%∼44% B; 45∼50 min, 44%∼45% B; 50∼55 min, 45%∼50% B; 55∼56 min, 50% B; 56∼61 min, 12% B. Flow rate: 0.8 mL·min^−1^; column temperature: 35°C; injection volume: 10 *μ*L; UV detection wavelength: 250 nm. On the basis of chromatographic conditions, all the components had good resolution.

### 2.7. Data Analysis

The data were analyzed and evaluated by Similarity Evaluation System for chromatographic fingerprint of TCM (Version 2004 A) which was recommended by the SFDA of China for evaluating similarities of chromatographic profiles of TCM. The similarity among different chromatograms was determined by calculating the correlative coefficient or cosine value of the vectorial angle [17–19]. HCA was carried out by calculating Squared Euclidean distance to distinguish preparation of different batches using SPSS. At the same time, we used the external standard method (ESM) and QAMS to calculate the 15 active components in 13 batches of SGS, respectively, to verify the feasibility of QAMS.

## 3. Results and Discussion

### 3.1. Chromatograph Optimization

**Figure 1:**
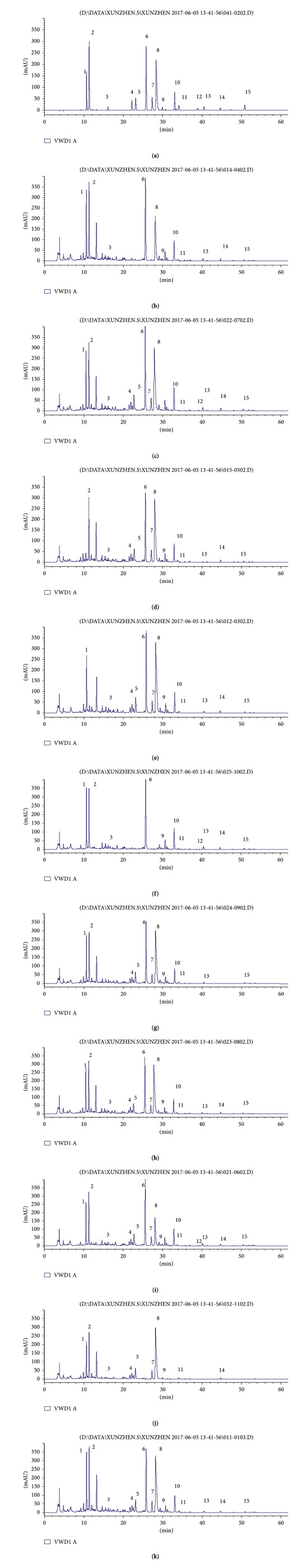
(a) Mixed standard solution, (b) negative sample without coptidis rhizoma, (c) negative sample without scrophulariae radix, (d) negative sample without anemarrhenae rhizome, (e) negative sample without gardeniae fructus, (f) negative sample without coptidis rhizoma and phellodendri chinensis cortex, (g) negative sample without glycyrrhizae radix et rhizoma preparata cum melle, (h) negative sample without cinnamomi cortex, (i) negative sample without phellodendri chinensis cortex, (j) negative sample without scutellariae radix, and (k) SGS sample. 1, mangiferin; 2, geniposide; 3, liquiritin; 4, epiberberine; 5, coptisine; 6, baicalin; 7, palmatine; 8, berberine; 9, harpagosid; 10, wogonoside; 10, cinnamic acid; 11, cinnamic aldehyde; 12, baicalein; 13, glycyrrhizic acid; and 14, wogonin.

At present, there are no single liquid phase conditions that can divide 15 components of SGS with good resolution. As the ingredients of SGS are very intricate, it is critical to establish a favorable mobile phase system, gradient elution system, and detection wavelength to obtain efficient separation of the numerous target components. The suitable ingredient of the HPLC method was investigated by checking peak resolution and the peak purity of SGS. In this case, some different mobile phases were tested which were acetonitrile-water, methanol-water, methanol-water containing phosphoric acid or formic acid at different concentrations, acetonitrile-water with acetic acid, formic acid, and phosphoric acid at different concentrations. Experimental results show that acetonitrile-water containing 0.2% phosphoric acid system produced sharp and symmetrical chromatographic peak shapes, good separation, and prevented the peak tailing. Chromatogram with the maximum number of peaks also relies on best conditions for preparation of sample solution. On the basis of the investigation of different solvent and ultrasonic time, it can be concluded that samples are dissolved in methanol and ultrasound 30 minutes; we can get better resolution and reproducibility of fingerprint chromatograms under the conditions of Section 2.6. Under the above chromatographic conditions, all the components were well separated (Figure 1).

### 3.2. Method Validation

#### 3.2.1. Linearity

A mixed solution containing all the reference substances were prepared and diluted in series with methanol to obtain six different concentrations. The different concentration of the mixed solution was used for constructing the reference curve. As shown in Table 2, good calibration curves of 15 compounds were obtained, and high correlation coefficient values (*R*^2^ > 0.999) were shown with good linearity at a wide range relatively. In response to sample concentration, the peak area of the analyte is determined by least squares linear regression to obtain a linear equation.

#### 3.2.2. Precision, Stability, Repeatability, and Recovery

The same mixed standard solution of 10 *µ*l was injected for six consecutive times under chromatographic conditions, and their RSDs were calculated. The RSD of mangiferin, geniposide, liquiritin, epiberberine, coptisine, baicalin, palmatine, berberine, harpagosid, wogonoside, cinnamic acid, cinnamic aldehyde, baicalein, glycyrrhizic acid, and wogonin was 1.94%, 0.72%, 0.88%, 0.54%, 0.62%, 0.97%, 0.93%, 1.35%, 0.98%, 1.33%, 0.67%, 1.40%, 1.08%, 0.96%, and 1.49% which indicated that the developed method had a good precision.

The stability of the sample solutions was analyzed at 0, 2, 4, 8, 12, and 24 h at room temperature. It was found that the sample solutions were stable within 24 h (RSD ≤ 5.0%).

To confirm the repeatability of the method, six independently prepared solutions from the same batch were analyzed. The RSD values of the peak area was 0.37%, 0.31%, 1.52%, 0.72%, 1.00%, 0.20%, 0.71%, 0.18%, 0.88%, 0.50%, 3.02%, 3.65%, 2.30%, 1.68%, and 3.23%, respectively. The results indicated the method is reproducible.

The recovery was performed by adding a known amount of individual standards into a certain amount of the SGS sample. The mixture was extracted and analyzed by using the method mentioned above. The average recoveries of 6 samples are shown in Table 3. The results show that the method is accurate. The recoveries of the 15 compounds which are shown in Table 3 ranged from 70.08% to 111.45% with RSDs ≤ 5.0%.

### 3.3. HPLC Fingerprint and Similarity Analysis

**Figure 2:**
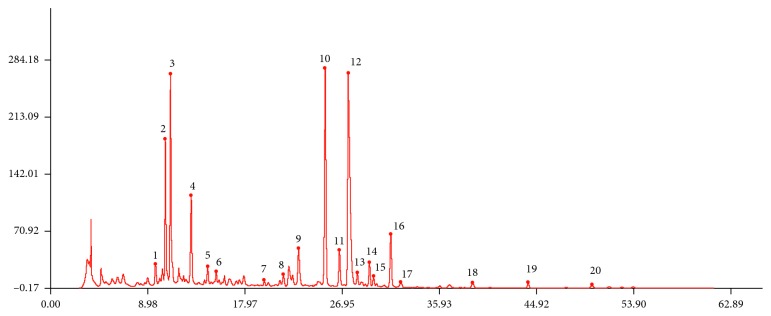
Antithesis fingerprint chromatogram of SGS.

**Figure 3:**
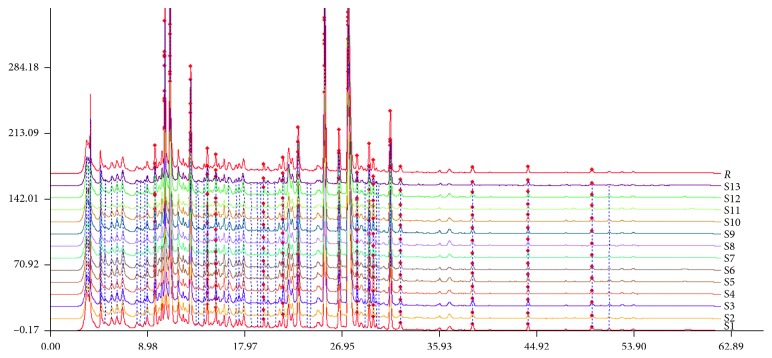
Fingerprint chromatograms of 13 batches of SGS.

**Figure 4:**
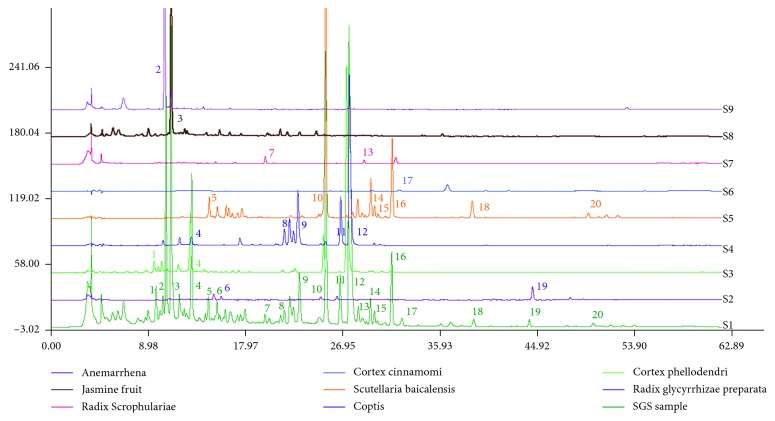
Comparison of single TCM pieces and SGS sample.

Thirteen batches of samples were prepared according to Section 2.5, and 10 *μ*L was injected into the HPLC system according to the chromatographic conditions given under Section 2.6, and then the chromatograms were recorded and entered into the similarity analysis software. We selected S (1) as the reference chromatogram, the utilization of the average correlation coefficient method of 13 batches of samples for multipoint correction, time window width is set to 0.5, while the establishment of a common model is to generate a control fingerprinting SGS, the antithesis fingerprint chromatogram was shown in Figure 2. Fingerprint chromatograms of 13 batches of SGS can be seen in Figure 3. As compared with the reference fingerprint chromatograms, the similarities of 13 batches of samples shown in Table 4, and the results are all above 0.95. On the basis of these results, we concluded that SGS between different batches are of good consistency and in line with the relevant requirements of the fingerprints. Palmatine is the main active ingredient of coptidis rhizoma; the corresponding peaks have favorable resolution, and the retention time is stable and moderate. Therefore, we selected palmatine (no. 11 peak) as the reference peak and calculated the relative retention time of the other common peaks. We can see that the retention time of the common peak is stable. According to the retention time of each fingerprint, a total of 20 common peaks were identified while 14 of them were determined. However, it should be pointed out that the chemical property of cinnamic aldehyde is very unstable due to its alkene structure of the molecule which has poor stability when exposed to light and oxygen, so it is not within the category of the common peaks [20]. This can be in accordance with S11 without the peak of cinnamic aldehyde. To gain better understanding of ascription of common peaks, reference standards and single TCM pieces were used. The peaks 2, 3, 6, 8, 9, 10, 11, 12, 13, 16, 17, 18, 19, and 20 were identified as mangiferin, geniposide, liquiritin, epiberberine, coptisine, baicalin, palmatine, berberine, harpagosid, wogonoside, cinnamic acid, cinnamic aldehyde, baicalein, glycyrrhizic acid, and wogonin, respectively (Figure 4). The peak 1 belongs to phellodendri chinensis cortex. The peak 4 belongs to both phellodendri chinensis cortex and coptidis rhizoma. The peaks 5, 14, and 15 belong to scutellariae radix. The peak 7 belongs to scrophulariae radix.

### 3.4. Hierarchical Cluster Analysis

**Figure 5:**
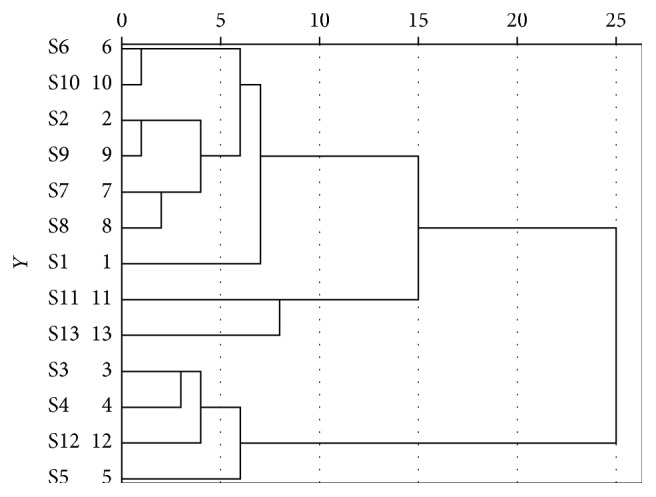
Clustering analysis graph of 13 SGS samples.

The 13 ^∗^ 20 matrices were obtained from 20 common peak areas of fingerprints of 13 batches of SGS. The cluster analysis was performed by using spss 2.0 software. The Euclidean distance was chosen as the measure of the distance between groups. The results are shown in Figure 5. S3, S4, S5, and S12 batches of samples are divided into a category; the remaining batches are divided into another classification, which indicates that there are differences in the content of the components in the samples prepared from different raw material TCM pieces. And it suggested that HCA was a valid method for the identification of the source of TCM pieces.

### 3.5. Quantitative Analysis of Multicomponents by a Single Marker (QAMS)

It is well established that many variations of experimental conditions, such as concentrations of standard, detector, and peak measurement parameters, would extremely influence the RCFs. Accordingly, the accuracy of RCFs may affect the final analysis results. Nevertheless, RCFs, which was calculated by linear-regression equation in the experiment, was considered to be accurate and stable [21, 22]. The RCFs were calculated using the calibration curves as follows:

(1) $$\begin{array}{c} F_{K/S}=\frac{a_{k}}{a_{s}}. \end{array}$$

The content of the measured component was calculated as follows:

(2) $$\begin{array}{c} C_{k}=\frac{A_{K}}{\left(A_{S}*F_{K/S}\right)}. \end{array}$$

*a*_s_ is the ratio of the slope of internal standard reference calibration equations; *a*_k_ is the ratio of the slope of measured component calibration equations; *A*_K_ is the peak area of the measured component; *A*_S_ is the peak area of the internal standard reference.

We investigated the influence of different instruments and different columns on the RCF values, and results are shown in Table 5 which illustrated RCF values had good repeatability on different chromatographic systems and different columns.

In this paper, we selected cheap, readily available, and chemically stable berberine as an internal reference standard for the quantitative determination of other active components. In addition to that, berberine is the main active ingredient of phellodendri chinensis cortex and coptidis rhizoma, so this study eventually takes it as an internal reference standard. The relative retention time has been used to locate target chromatographic peaks.

(3) $$\begin{array}{c} t_{k/s}=\frac{t_{k}}{t_{s}}, \end{array}$$

where *t*_s_ is the retention time of internal standard reference and *t*_k_ is the retention time of measured component.

The internal reference is berberine; the relative retentions between the other target peaks and berberine were obtained in different columns and HPLC instruments. Results are shown in Table 6. The results showed that their RSDs ≤ 5% and no interference with other components; the relative retention time can be applied to locate the peak component of the analytes.

**Figure 6:**
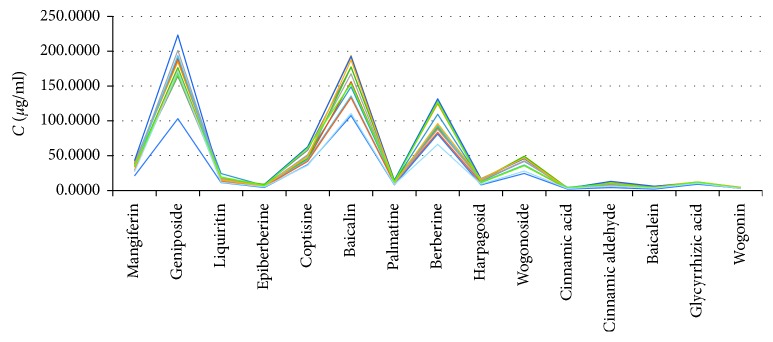
The contents of 15 active components in 13 batches of SGS.

We measured the multicomponent content of 13 batches SGS (Figure 6); the results showed that there were significant differences in some contents of 15 ingredients, such as cinnamic aldehyde and baicalein, which indicated that only a few ingredients of the standard determination of content could not control the quality of SGS effectively. It is necessary to use multiple active ingredients as index components to control the quality of TCM preparations more comprehensively.

To validate the difference between ESM and QAMS method using RCFs, 13 SGS samples were analyzed for their active ingredients. The calculated results are shown in Table 7. Standard method difference (SMD) is calculated according to the following equation:

(4) $$\begin{array}{c} \text{SMD}=\frac{\left(C_{\text{ES}}-C_{\text{QAMS}}\right)}{C_{\text{ES}}}*100\%, \end{array}$$

where *C*_ES_ and *C*_QAMS_ represent the concentrations of an analyte assayed by the external standard method and QAMS method, respectively [23]. All the values of standard deviation (SMD < 0.05) revealed that there were no significant differences between ESM and QAMS methods of all SGS samples.

## 4. Conclusion

On the basis of these results, we concluded that HPLC fingerprint method based on chemical constituents profiling was an effective and stable tool, and QAMS method was feasible to quantify the active compounds by RCFs for evaluating the quality of SGS. Along with similarity analysis and HCA of synthesis, the quality of SGS would be evaluated and better identified comprehensively. This method could potentially be applied in the quality control of TCM.

## Figures and Tables

**Table 1:** Species and geographical locations of eight Chinese herbal pieces in SGS.

Botanical name	Family	Collection site	Coordinates	Voucher ID
*Coptis chinensis* Franch.	Ranunculaceae	Sichuan	N30°15′49.6902″ S102°48′19.71″	16121204

*Phellodendron chinense* Schneid.	Rutaceae	Sichuan	N30°15′49.6902″ S102°48′19.71″	1508120316060500

*Scutellaria baicalensis* Georgi	Labidae	Heilongjiang	N47°7′17.9292″ S128°44′17.6316″	160401

*Scrophularia ningpoensis* Hemsl.	Scrophulariaceae	Yunnan	N24°28′31.0254″ S101°20′35.1816″	16060501

*Anemarrhena asphodeloides* Bge.	Liliaceae	Jiangsu	N33°8′24.6186″ S119°47′20.13″	16032208

*Gardenia jasminoides* Ellis	Rubiaceae	Jiangxi	N27°5′14.841″ S114°54′15.1956″	16122107

*Cinnamomum cassia* Presl.	Aceraceae	Sichuan	N30° 15′ 49.6902″ S102° 48′ 19.71″	16112519

*Glycyrrhiza uralensis* Fisch.	Legume	Neimenggu	N43°22′41.5914″ S115°3′34.1316″	16021710

*Gypsum fibrosum*	Monoclinic crystal system	Hunan	N27°37′31.0794″ S111°51′24.6924″	16120420

**Table 2:** Standard curves of fifteen kinds of reference components.

Compounds	Regression equations	Linear ranges (*μ*g·mL^−1^)	*R* ^2^
Mangiferin	*y* = 29.897*x* + 30.359	2.80–88.70	0.9998
Geniposide	*y* = 11.306*x* − 40.123	14.80–472.90	0.9997
Liquiritin	*y* = 6.9865*x* − 5.2633	3.20–101.00	1.0000
Epiberberine	*y* = 23.477*x* − 12.998	1.40–44.30	0.9998
Coptisine	*y* = 11.677*x* − 31.945	6.40–204.10	0.9998
Baicalin	*y* = 17.312*x* − 189.17	19.80–632.50	0.9999
Palmatine	*y* = 39.570*x* − 21.105	1.80–56.80	0.9999
Berberine	*y* = 38.357*x* + 108.6	15.30–488.40	0.9999
Harpagosid	*y* = 9.5715*x* + 8.4144	2.60–82.60	0.9998
Wogonoside	*y* = 16.462*x* + 11.105	4.60–145.82	0.9999
Cinnamic acid	*y* = 32.7*x* + 0.4639	0.30–9.51	0.9998
Cinnamic aldehyde	*y* = 15.523*x* − 0.1567	0.20–6.34	0.9998
Baicalein	*y* = 25.996*x* + 3.789	0.50–15.85	0.9998
Glycyrrhizic acid	*y* = 7.5138*x* + 1.333	1.10–34.00	0.9999
Wogonin	*y* = 21.652*x* + 0.8509	0.30–9.51	1.0000

**Table 3:** The results of recovery of fifteen components in samples (*n*=6).

Compound	Original (mg)	Added amount (mg)	Detected amount (mg)	Recovery (%)	RSD (%)
Mangiferin	0.0241	0.0241	0.0478	93.96	1.18
Geniposide	0.1036	0.1036	0.2032	99.58	2.60
Liquiritin	0.0198	0.0198	0.0380	96.11	3.42
Epiberberine	0.0073	0.0073	0.0142	103.46	3.61
Coptisine	0.0223	0.0223	0.0438	110.19	3.16
Baicalin	0.1226	0.1226	0.2139	81.82	2.61
Palmatine	0.0104	0.0104	0.0200	97.21	2.28
Berberine	0.0748	0.0748	0.1539	101.87	5.31
Harpagosid	0.0212	0.0212	0.0433	100.01	3.56
Wogonoside	0.0307	0.0307	0.0620	99.81	3.33
Cinnamic acid	0.0023	0.0023	0.0048	111.45	2.38
Cinnamic aldehyde	0.0023	0.0023	0.0040	70.08	5.00
Baicalein	0.0048	0.0048	0.0095	79.84	3.73
Glycyrrhizic acid	0.0079	0.0079	0.0155	94.08	2.52
Wogonin	0.0028	0.0028	0.0056	98.34	4.19

**Table 4:** Similarities of 13 batches SGS.

	S1	S2	S3	S4	S5	S6	S7	S8	S9	S10	S11	S12	S13	*R*
S1	1	0.984	0.984	0.982	0.989	0.981	0.991	0.99	0.978	0.984	0.982	0.987	0.978	0.994
S2	0.984	1	0.975	0.978	0.985	0.986	0.985	0.995	0.997	0.987	0.979	0.973	0.994	0.994
S3	0.984	0.975	1	0.991	0.987	0.978	0.974	0.974	0.964	0.983	0.995	0.995	0.959	0.991
S4	0.982	0.978	0.991	1	0.991	0.99	0.98	0.975	0.972	0.99	0.987	0.988	0.96	0.992
S5	0.989	0.985	0.987	0.991	1	0.985	0.992	0.989	0.98	0.985	0.988	0.988	0.975	0.996
S6	0.981	0.986	0.978	0.99	0.985	1	0.983	0.982	0.985	0.993	0.972	0.972	0.974	0.992
S7	0.991	0.985	0.974	0.98	0.992	0.983	1	0.992	0.983	0.98	0.975	0.979	0.983	0.992
S8	0.99	0.995	0.974	0.975	0.989	0.982	0.992	1	0.994	0.982	0.977	0.975	0.993	0.994
S9	0.978	0.997	0.964	0.972	0.98	0.985	0.983	0.994	1	0.985	0.969	0.963	0.994	0.989
S10	0.984	0.987	0.983	0.99	0.985	0.993	0.98	0.982	0.985	1	0.977	0.977	0.973	0.993
S11	0.982	0.979	0.995	0.987	0.988	0.972	0.975	0.977	0.969	0.977	1	0.994	0.966	0.99
S12	0.987	0.973	0.995	0.988	0.988	0.972	0.979	0.975	0.963	0.977	0.994	1	0.962	0.99
S13	0.978	0.994	0.959	0.96	0.975	0.974	0.983	0.993	0.994	0.973	0.966	0.962	1	0.985
*R*	0.994	0.994	0.991	0.992	0.996	0.992	0.992	0.994	0.989	0.993	0.99	0.99	0.985	1

**Table 5:** RCFs of berberine to mangiferin, geniposide, liquiritin, epiberberine, coptisine, baicalin, palmatine, harpagosid, wogonoside, cinnamic acid, cinnamic aldehyde, baicalein, glycyrrhizic acid, and wogonin on different instruments and different columns.

Instrument	Column	Mangiferin	Geniposide	Liquiritin	Epiberberine	Coptisine	Baicalin	Palmatine	Harpagosid	Wogonoside	Cinnamic acid	Cinnamic aldehyde	Baicalein	Glycyrrhizic acid	Wogonin
Waters2695-2998	Amethy	0.7632	0.3112	0.1888	0.6033	0.3144	0.4421	1.0451	0.2561	0.4321	0.8611	0.4043	0.6811	0.1993	0.5532
	Hedra	0.7794	0.3008	0.1791	0.6215	0.3211	0.4351	1.0733	0.2671	0.4411	0.8835	0.4156	0.6632	0.1911	0.5783
	Agilent	0.7794	0.2948	0.1821	0.6121	0.3044	0.4513	1.0316	0.2495	0.4292	0.8525	0.4047	0.6777	0.1959	0.5645
Agilent1100	Amethy	0.7553	0.3138	0.1813	0.5988	0.3198	0.4579	1.0651	0.2355	0.4351	0.8421	0.4255	0.6843	0.2021	0.5547
	Hedra	0.7421	0.3097	0.1923	0.5899	0.3176	0.4621	1.0688	0.2466	0.4284	0.8311	0.4322	0.6731	0.2124	0.5832
	Agilent	0.7342	0.3201	0.1799	0.6031	0.3021	0.4633	1.0803	0.2611	0.4511	0.8941	0.4167	0.6864	0.2145	0.5401
Mean		0.7589	0.3084	0.1839	0.6048	0.3132	0.4520	1.0607	0.2527	0.4362	0.8607	0.4165	0.6776	0.2025	0.5623
RSD (%)		2.48	2.97	2.91	1.80	2.58	2.52	1.75	4.45	1.98	2.81	2.67	1.26	4.56	2.90

**Table 6:** Rentention time (min) of berberine to mangiferin, geniposide, liquiritin, epiberberine, coptisine, baicalin, palmatine, harpagosid, wogonoside, cinnamic acid, cinnamic aldehyde, baicalein, glycyrrhizic acid, and wogonin on different instruments and different columns.

Instrument	Column	Mangiferin	Geniposide	Liquiritin	Epiberberine	Coptisine	Baicalin	Palmatine	Harpagosid	Wogonoside	Cinnamic acid	Cinnamic aldehyde	Baicalein	Glycyrrhizic acid	Wogonin
Waters2695-2998	Amethy	0.38	0.40	0.58	0.80	0.83	0.92	0.97	1.04	1.14	1.17	1.34	1.41	1.60	1.81
	Hedra	0.41	0.43	0.62	0.82	0.84	0.96	0.97	1.06	1.11	1.18	1.28	1.54	1.59	2.02
	Agilent	0.39	0.41	0.60	0.81	0.83	0.94	0.97	1.05	1.13	1.17	1.30	1.48	1.60	1.91
Agilent1100	Amethy	0.36	0.39	0.57	0.75	0.81	0.90	0.95	1.02	1.13	1.16	1.30	1.38	1.59	1.79
	Hedra	0.37	0.41	0.58	0.77	0.83	0.92	0.96	1.05	1.12	1.16	1.33	1.40	1.58	1.78
	Agilent	0.40	0.42	0.60	0.82	0.85	0.94	0.99	1.06	1.16	1.19	1.36	1.43	1.62	1.83
Mean		0.39	0.41	0.59	0.79	0.83	0.93	0.97	1.05	1.13	1.17	1.32	1.44	1.60	1.86
RSD (%)		5.00	3.03	3.38	4.03	2.76	2.66	1.90	1.85	2.32	1.77	2.95	4.33	1.95	5.00

**Table 7:** Comparison of the results from the ESM and QAMS (*μ*g·ml^−1^).

Batch number	Berberine	Mangiferin		Geniposide		Liquiritin		Epiberberine		Coptisine		Baicalin		Palmatine	
	ESM	QAMS	ESM	QAMS	ESM	QAMS	ESM	QAMS	ESM	QAMS	ESM	QAMS	ESM	QAMS	ESM
1	109.5341	40.2024	40.2261	181.4930	189.7332	23.0219	24.3703	6.3113	7.0281	46.8326	50.7789	134.5759	148.9816	11.4799	12.3100
2	89.2249	37.5741	37.7510	179.5150	188.7602	15.1217	16.3549	5.8212	6.5595	40.4232	44.4416	140.9748	156.3753	10.5081	11.3749
3	129.1133	35.8398	35.6103	157.0895	164.0831	15.6869	16.7843	7.5026	8.2207	54.0515	57.9725	163.0149	177.5168	12.7851	13.5988
4	124.5509	39.7362	39.6241	177.6354	185.2223	16.2344	17.3568	8.0797	8.8171	57.6934	61.7406	178.8141	193.8060	13.3434	14.1801
5	131.7465	42.8953	42.8017	215.0841	223.2552	13.4517	14.4941	8.1313	8.8596	58.7696	62.7683	177.6743	192.4197	13.8791	14.7108
6	91.8581	33.9407	33.9713	167.7467	176.4659	13.4688	14.6373	8.0163	8.8171	41.8712	45.8975	161.3843	177.2857	13.0916	14.0284
7	93.6830	33.7007	33.7037	184.5859	193.7133	12.6430	13.7785	5.6230	6.3466	40.0668	44.0134	121.2770	135.8693	10.4254	11.2738
8	83.4633	28.7922	28.7534	161.0843	170.0976	11.7671	12.9197	4.9025	5.6224	37.9355	41.9581	118.3847	133.3277	9.5326	10.3893
9	87.8692	35.7417	35.8779	191.4261	201.1430	15.2532	16.4980	5.3645	6.0910	47.1242	51.3784	151.7083	167.5237	9.7197	10.5662
10	96.1076	33.3683	33.3358	163.7588	172.1319	13.7647	14.9235	5.8754	6.6021	44.4223	48.4666	171.5295	187.5098	10.4331	11.2738
11	81.0647	21.1692	20.8931	96.3176	103.2304	10.2344	11.3452	3.7042	4.3872	33.2648	37.1624	93.3769	107.5653	7.3989	8.1907
12	127.4969	35.4701	35.2424	160.0752	167.1788	18.2031	19.3607	6.7088	7.4114	56.9690	60.9699	139.2935	153.3139	11.8422	12.6385
13	66.2826	26.8813	27.0141	161.2528	171.6896	10.7070	11.9177	4.4526	5.1965	32.0308	36.1347	95.5600	110.5690	7.9011	8.7719
*p* value		1.0000		0.9990		1.0000		1.0000		1.0000		1.0000		1.0000	
SMD		−0.14%		4.84%		5.00%		4.40%		3.88%		4.64%		5.00%	

Harpagosid		Wogonoside		Cinnamic acid		Cinnamic aldehyde		Baicalein		Glycyrrhizic acid		Wogonin			
QAMS	ESM	QAMS	ESM	QAMS	ESM	QAMS	ESM	QAMS	ESM	QAMS	ESM	QAMS	ESM		

14.2582	13.7492	41.5099	41.9147	4.3225	4.4343	10.1104	10.3781	3.8998	3.8544	10.7680	10.8733	2.8363	2.8704		
14.3794	13.9581	41.7443	42.4007	3.3197	3.4251	8.1171	8.3811	4.2131	4.2006	10.3196	10.4741	3.3573	3.4246		
13.6995	13.1223	42.3230	42.5829	3.0224	3.0887	8.5102	8.7032	5.2322	5.2008	11.9814	12.0711	3.6155	3.6556		
14.3017	13.7492	48.7639	49.2042	3.6181	3.7003	10.8341	11.0868	6.0932	6.0856	12.1021	12.2042	4.6062	4.6716		
17.3874	16.8835	45.4335	45.7417	3.2033	3.2722	12.8653	13.1482	6.2136	6.2010	10.8139	10.8733	4.1144	4.1636		
15.7097	15.3163	48.3223	49.1435	3.4117	3.5168	6.2494	6.4485	4.9259	4.9315	9.4250	9.5424	3.8532	3.9327		
12.4737	11.9730	36.3218	36.7513	2.9684	3.0581	7.2536	7.4792	3.2858	3.2390	11.1098	11.2726	2.7346	2.7780		
11.7217	11.2417	35.2517	35.7794	2.6324	2.7217	9.3460	9.6695	3.3857	3.3544	9.5254	9.6755	2.8142	2.8704		
14.9799	14.5850	41.7833	42.4614	3.9107	4.0367	7.6140	7.8657	4.4720	4.4699	10.9594	11.1395	4.2506	4.3483		
16.8469	16.4655	46.1440	46.8351	2.4359	2.5076	5.3190	5.4822	4.5214	4.5084	12.1523	12.3373	4.0377	4.1174		
8.5808	8.0029	24.3588	24.5414	1.9207	1.9878	4.0460	4.1938	2.0815	2.0080	8.7446	8.8770	3.2131	3.2861		
11.9582	11.3462	35.5964	35.7186	3.8293	3.9144	9.8943	10.1205	3.9137	3.8544	12.1083	12.2042	3.5242	3.5632		
9.5187	9.0477	27.3810	27.8824	2.6982	2.8135	5.8692	6.1264	3.3202	3.3159	10.0833	10.3410	2.9233	3.0090		
1.0000		1.0000		1.0000		1.0000		1.0000		0.9990		1.0000			
−4.00%		1.15%		2.84%		2.92%		−0.80%		1.28%		1.75%			

## Data Availability

The datasets used or analyzed during the current study are available from the corresponding author on reasonable request. All data generated or analyzed during this study are included in this submitted manuscript.
